# Supplementary material for: The Endo-α(1,3)-Fucoidanase Mef2 Releases Uniquely Branched Oligosaccharides from Saccharina latissima Fucoidans
Source: Mar Drugs. 2022 Apr 29;20(5):305. doi: 10.3390/md20050305 (PMC9147238; doi:10.3390/md20050305)
Supplement: Supplementary file 1 [file marinedrugs-20-00305-s001.zip › marinedrugs-1687240-supplementary.pdf]

Supplementary Information

Table S1. Percent identity matrix of the catalytic D1 domain of Mef2 and the characterized members of the GH107 family

	MfFcnA	FFA1	Fhf1	FFA2	FWf1	FWf3	P5AFcnA	P19DFcnA	**Mef2**	Fda1	Fda2	SVI_0379
MfFcnA/34-418*	*100*	83	72	63	53	38	16	17	14	23	23	26
FFA1/31-415*	83	*100*	72	62	54	37	16	17	14	23	23	25
Fhf1/29-447*	72	72	*100*	68	54	38	17	17	16	25	24	25
FFA2/41-433*	63	62	68	*100*	52	39	17	17	16	23	22	25
FWf1/15-439*	53	54	54	52	*100*	40	17	16	13	24	25	23
FWf3/213-589*	38	37	38	39	40	*100*	15	18	15	23	24	22
P5AFcnA/36-393*	16	16	17	17	17	17	*100*	72	39	22	23	22
P19DFcnA/31-393*	17	17	17	17	16	18	72	*100*	40	23	24	22
**Mef2**/22-383*	14	14	16	16	13	15	**39**	**40**	*100*	**21**	**23**	20
Fda1/23-372*	23	23	25	23	24	23	22	23	21	*100*	72	44
Fda2/84-439*	23	23	24	22	25	24	23	24	23	72	*100*	40
SVI_0379/26-367*	26	25	25	25	23	22	22	22	20	44	40	*100*

* Indicate the amino acid sequence numbers used for the analysis

Table S2. Thermostability by DLS of Mef2 with Ca^{2+} and without Ca^{2+}

**Mef2 Stability**	**Tm (°C)**
Mef2 - Ca^{2+}	37.9 ± 0.05
Mef2 + Ca^{2+}	44.4 ± 0.03

Table S3. Monosaccharide composition of fucoidan fraction 4 (FeF4) purified from enzymatic extracts of *F. evanescence*

		**FeF4**
Neutral monosaccharides (mol%)	Fucose	88.1 ± 0.0
	Rhamnose	0.8 ± 0.0
	Galactose	7.3 ± 0.3
	Glucose	0.7 ± 0.1
	Xylose	1.1 ± 0.1
	Mannose	0.2 ± 0.0
Uronic acids (%)	Guluronic acid	0.1 ± 0.1
	Glucuronic acid	0.5 ± 0.2
	Mannuronic acid	1.1 ± 0.5
Sulfate (%)		38.1 ± 1.2

The data are given in %mol (relative level) of total carbohydrates analyzed in the fucoidan fractions

Table S4. Monosaccharide composition of fucoidan fractions purified from enzymatic extracts of *S. latissima* from Ocean Rainforest

		SlF1	SlF2	SlF3	SlF4
Neutral monosaccharides (mol%)	Fucose	$31.7^{c} \pm 2.2$	$69.0^{a} \pm 0.8$	$58.6^{b} \pm 0.3$	$63.1^{b} \pm 0.5$
	Rhamnose	$0.4^{c} \pm 0.1$	$1.0^{b} \pm 0.0$	$0.8^{b,c} \pm 0.0$	$2.0^{a} \pm 0.1$
	Galactose	$0.5^{d} \pm 0.1$	$17.4^{c} \pm 0.2$	$34.8^{a} \pm 0.4$	$22.1^{b} \pm 0.0$
	Glucose	$0.3^{d} \pm 0.0$	$0.4^{c} \pm 0.0$	$0.5^{b} \pm 0.0$	$0.9^{a} \pm 0.0$
	Xylose	$2.4^{c} \pm 0.3$	$5.1^{b} \pm 0.2$	$2.7^{c} \pm 0.1$	$7.3^{a} \pm 0.3$
	Mannose	$0.6^{d} \pm 0.1$	$2.1^{a} \pm 0.1$	$1.0^{c} \pm 0.0$	$1.8^{b} \pm 0.0$
Uronic acids (%)	Guluronic acid	$2.5^{a} \pm 0.1$	$0.2^{b} \pm 0.1$	$0.1^{b} \pm 0.0$	$0.2^{b} \pm 0.0$
	Glucuronic acid	$1.3^{b,c} \pm 0.3$	$3.7^{a} \pm 0.4$	$1.1^{c} \pm 0.0$	$2.3^{b} \pm 0.0$
	Mannuronic acid	$60.2^{a} \pm 3.2$	$1.2^{b} \pm 0.1$	$0.4^{b} \pm 0.3$	$0.5^{b} \pm 0.1$
Sulfate (%)		$11.3^{c} \pm 1.0$	$28.4^{b} \pm 2.1$	$35.6^{a} \pm 0.4$	$32.1^{a} \pm 3.1$

The data are given in %mol (relative level) of total carbohydrates analyzed in the fucoidan fractions
Different superscript letters a,b,c indicate statistically different values ($p < 0.05$) between the values in the fucoidan fractions

Table S5. Monosaccharide composition of *S. latissima* MMP after Mef2 hydrolysis

		MMP
Neutral monosaccharides (mol%)	Fucose	48.1 ± 0.0
	Rhamnose	1.0 ± 0.6
	Galactose	44.0 ± 3.7
	Glucose	0.3 ± 0.2
	Xylose	2.3 ± 0.3
	Mannose	1.7 ± 1.9
Uronic acids (%)	Guluronic acid	1.6 ± 1.2
	Glucuronic acid	0.7 ± 0.1
	Mannuronic acid	0.3 ± 0.5
Sulfate (%)		34.2 ± 1.8

The data are given in %mol (relative level) of total carbohydrates analyzed in the fucoidan fractions

Table S6. Monosaccharide composition and sulfate content of fractionated *S. latissima* MMP

Fractions	Monosaccharide composition Fuc:Gal:Rha:Glu:Xyl:Man:GuluA:GluA:ManA (mol:mol)	Sulfate, %
MF1	1 : 0.29 : 0.07: 0.02 : 0.14 : 0.02: 0.00: 0.02: 0.02	52.1 ± 0.8
MF2	1 : 0.42 : 0.06: 0.02 : 0.12 : 0.02: 0.00: 0.01: 0.02	48.2 ± 1.9
MF3	1 : 0.56 : 0.04: 0.02 : 0.07 : 0.01: 0.00: 0.01: 0.01	52.1 ± 0.8
MF4	1 : 0.52 : 0.03: 0.02 : 0.06 : 0.01: 0.00: 0.00: 0.01	54.8 ± 2.9
MF5	1 : 0.73 : 0.03: 0.02 : 0.07 : 0.01: 0.00: 0.00: 0.01	27.8 ± 1.6
MF6	1 : 0.65 : 0.03: 0.02 : 0.06 : 0.01: 0.00: 0.00: 0.02	28.7 ± 3.3

Table S7. Monosaccharide composition of fucoidan fraction 3 from different brown seaweed species

		S. polycystum	*S. oligocystum*	*S. serratum*	*T. ornata*	*H. cuneiformis*	*S. mcclurei*
Neutral monosaccharides (%mol)	Fucose	46.7[d] ± 4.5	66.4[c] ± 3.4	81.5[a,b] ± 0.8	85.6[a] ± 0.3	72.7[b,c] ± 0.0	78.5[a,b] ± 1.7
	Rhamnose	0.5[a] ± 0.4	0.8[a] ± 0.6	0.2[a] ± 0.0	0.0 ± 0.0	0.3[a] ± 0.4	0.3[a] ± 0.0
	Galactose	26.1[a] ± 4.9	24.3[a,b] ± 2.3	9.7[c] ± 0.4	9.9[c] ± 0.5	20.2[a,b] ± 0.4	15.4[b,c] ± 0.5
	Glucose	1.3[a] ± 0.0	0.7[b] ± 0.3	0.2[b,c] ± 0.0	0.2[b,c] ± 0.0	0.2[c] ± 0.1	0.1[c] ± 0.1
	Xylose	18.3[a] ± 2.6	3.8[b] ± 0.1	3.7[b] ± 0.2	0.8[b] ± 0.0	2.6[b] ± 0.1	2.8[b] ± 0.3
	Mannose	0.0 ± 0.0	1.1[a] ± 0.0	0.6[b] ± 0.0	0.20[c] ± 0.1	0.0 ± 0.0	0.0 ± 0.0
Uronic acids (%mol)	Guluronic acid	0.7[a] ± 0.2	0.1[a] ± 0.0	0.1[a] ± 0.0	0.2[a] ± 0.0	0.4[a] ± 0.2	0.1[a] ± 0.1
	Glucuronic acid	0.6[a] ± 0.1	1.0[a] ± 0.1	0.5[a] ± 0.0	0.5[a] ± 0.2	1.0[a] ± 0.4	0.5[a] ± 0.2
	Mannuronic acid	5.2[a] ± 0.5	1.8[a] ± 0.0	3.4[a] ± 0.1	2.5[a] ± 0.0	2.4[a] ± 0.3	2.0[a] ± 2.1
Sulfate (%)		24.6[c] ± 0.8	27.7[c] ± 1.8	38.1[a] ± 4.0		29.4[b,c] ± 2.3	31.4[b] ± 2.6

The data are given in %mol (relative level) of total carbohydrates analyzed in the fucoidan fractions
Different superscript letters a,b,c indicate statistically different values ($p < 0.05$) between the values in the fucoidan samples

```
FWf3/213-589         ALGERAVNLRGTWN-------------SEI------LVDLLDDRIKNLKTLNRLGVSFTN    41
Fp273/234-617        AAGERAVGLRGTWV-------------GGV------GD---LESIKHFKTLTTMGASFSS    38
FFA2/41-433          DLGLRADWMRGSLGLLWLPENNYNGNIEGV------SIEDFLTQIQHLKTVDFVQVGLAS    54
Fhf2/31-439          DLGLRADWMRGSLGLLWLPENNHNGNIEGV------SIEAFLTQIQHLKTVDFIQVGLAS    54
Fhf1/29-447          NQGLRANWMRGSWAALWLPENNYNGHIEGV------SIEPFIEQIKELETIDYIQIGLTN    54
FWf4/37-421          EQGLRANWMRGALGLLWLPERNYNGNIEGV------EIDEFISQVKDLRTIDYVQLPLTS    54
MfFcnA/34-418        NQGLRAEWMRGALGMLWLPERTFNGNIEGI------RIDDFLTQIKDIRTVDYVQLPLTS    54
FFA1/31-415          DQGLRAEWMRGSYGMLWLPERNFNGNIEGI------RIDEFITQIKDIRTVDYVQLPLTS    54
FWf2/243-679         ALGERADWLRGTWGVTWTPSNYQNGNVEDV------SIDDFLNQISEVKTISYIQVKLGS    54
Fp279/40-449         KITTRADWLRGSWGLNWKPTDLWNGAGEAL------TIDLFLEQISHLKTIDYVQVHLNE    54
FWf1/15-439          NQGLRAHWLRGTWGINWKPVNLYNGGHEGL------SIEPFLNQISHIKTIDYIQVHLGE    54
Fp277/26-435         -VESRGDWLRNSWGINWKPVDVYNGKSESL------EIDIFLDQISHLKTIDYIQSHLGE    53
SVI_0379/26-367      -TESKAVWMEGKVGVGFRINADR----KD-NIENY-DVDTLVNQIKTIDGISYVIFNLSD    53
Fda1/23-372          --ETKADWMQGNWGISYRIPGGDINYSGS-HVAEY-NVRAAVEQISAIPGLKWVQINLTN    56
Fda2/84-439          PEDIRASWMQGNWGISFRISGGDISQNES-HVNEY-QVAPAVEQIAAIPGLKWLQVNLSN    58
Mef2/22-383          ----DDSWLEGSWGITFPVFGGARLDAEV--AGGLDPVSGAQEIVDELSEVGHVITNLSY    54
P5A_FcnA/36-393      GIEYNASWMAGTWGITQRVDGGYKLDNSA---DSSNWQAGAEEIVTNIPAAEYVITSFTH    57
P19D_FcnA/31-393     DSEYTANWMAGAWGITQRVDGGYKL-DASVETGKYDWVAGAEEIVENIPSAGYVITSFTH    59
                             : .                                 :  .     :   :

FWf3/213-589         GAVLSAEHKVPHKLFDRLSG-------------GDVVAPSWGDL--NSDTPDNDNMLADI    86
Fp273/234-617        GASASAQHNAPHKFLETIALKGV---NGNGPAIDYVVAPSWGDL--SLDEPESDPFLDNI    93
FFA2/41-433          PYIYSPVHTAPHPLLERLWEGDL---GPDGKPI-NLVVPRASA---------PDPFLGWL    101
Fhf2/31-439          PYIYSPVHTAPHPLLEKLWEGDL---GPDGKPI-NLVVPRASA---------PDPFLSWL    101
Fhf1/29-447          PNIYSPVHTAPHPIIESLWQGDV---DPDGNPL-NLVVPRASA---------PDPFLSWL    101
FWf4/37-421          PNIFSPVHVAPHSIIESLWEGDT---DSNGDPI-NLVVPRESV---------DDPLLRWL    101
MfFcnA/34-418        PNIFSPTHVAPHPIIESLWQGDT---DANGDPI-NLVAPRESV---------DDPLLSWL    101
FFA1/31-415          PNIFSPIHVGPHSLIESFWEGDT---DANGDPI-NLAVPRESV---------DDPLLSWL    101
FWf2/243-679         SYIYSPVFAAPHQVLESFWYNDGVDRDGNGEPVQNLVVPRWGNG--TI--AENDPFLDWI    110
Fp279/40-449         SYINSPSHLGTHELLESFWQGDT---SPLGTPM-NLVVPRAST--------GVDPFLEMI    102
FWf1/15-439          SSIKSSVHMGPHSLLESFWEGDT---DANGDPI-NLVVPRASY--------GEDPFLEIV    102
Fp277/26-435         SSIKSSVHIAPHELLESFWQGDT---DATGNPV-NLVVPRASA--------NVDPFLKIL    101
SVI_0379/26-367      AAH-GDAYLAPHSIL---------------TTLNPDSTPNAD---------RDLFGELA     87
Fda1/23-372          GAS-GDRFIVPVTEV---------------EAINPLSAPNSINDLYDPTLPGRDLFEQLA    100
Fda2/84-439          GAF-GDRFIVPVPEV---------------EAINPNSAPNSSADLFDPALPGDDLFEQIA    102
Mef2/22-383          FAH-SHYFTLSQNNYINIR-----------NEIHPDVVP---------SAANDDIIFDVL    93
P5A_FcnA/36-393      PAH-GHLFTLRTNNNVDV------------SAIHPDMVP---------TLENEKIILDVI    95
P19D_FcnA/31-393     PAH-GFLYTLRDNENVDV------------AAIHPDMVP---------SLENEKIIFDVI    97
                         .  .                             .*              . :

FWf3/213-589         LKIKAAGFNVKAYTNSENFVGT--------------------------------------    108
Fp273/234-617        LAIKAAGFKIKAYLNSENFLGS--------------------------------------    115
FFA2/41-433          KAIKAAGLKTEVYVNSANLLEWEAF------G----------------------------    127
Fhf2/31-439          KAIKAAGLKTEVYVNSANLLQWEAF------N----------------------------    127
Fhf1/29-447          KAIKAAGLKTEIYVNSYNLLARVP-------D----------------------------    126
FWf4/37-421          KALKAEGLRVEIYVNSYNLLARNP-------E----------------------------    126
MfFcnA/34-418        KALRAAGLRTEIYVNSYNLLARIP-------E----------------------------    126
FFA1/31-415          KALRAAGLKAEIYVNSYNLLARIP-------E----------------------------    126
FWf2/243-679         TKIKAAGLKVQVYVNSSNMLQRYSDSYEDGRI----------------------------    142
Fp279/40-449         KKSQKAGLKVQVYVNSSNLLTRG-A-----TF----------------------------    128
FWf1/15-439          KAIRAAGLKVMVYVNSSNMLSREGP-----GG----------------------------    129
Fp277/26-435         KKVKAAGMKNQVYVNSSNMLQNES-------T----------------------------    126
SVI_0379/26-367      KAFHAAGIKVIAYAATQGPAML-----KHGAAYAFDGVQD--------------------    122
Fda1/23-372          LAFKAKGIRVVAYIATQGPGML-----KHGAENSMDEDDSIT------DCKSSKPLVTDL    149
Fda2/84-439          LGLQAKGIKVVAYIATQGPAML-----KHGAERSMDFDDSIVDESDGSACKSSRPVVSDP    157
Mef2/22-383          DIFKNSDKKIILYISTQYFAR------ADEDN----------------------------    119
P5A_FcnA/36-393      NIYRAAGKKVILYLNSAGPSMA-----EERGD----------------------------    122
P19D_FcnA/31-393     NVYKSAGKKVLLYLNTAGPTHA-----ADRNS----------------------------    124
                        :  . .   *  :

FWf3/213-589         -NADYLQPFVDSWKEYCDTDPEVQAFINSQPYHTGIWNRTTEQYEDATATYPNRKYMFCY    167
Fp273/234-617        -NQDHLQEFVDRWKEYCDNDAVVQAFINSQPFHTSVWDTETQTYVDATETYPNRKYMFAY    174
FFA2/41-433          TPISEFPFDFSERWKAYCDTDPTMQAFIESQPYHKDGVN-----------DDRRPYMFCY    175
Fhf2/31-439          GAPSEFPFDFSNRWKAYCDTDPTVQAFINSEAYHKDGVN-----------DDRRPYMFAY    175
Fhf1/29-447          GIPAGYPDLSERWEEYCNTNATAQAFIKNNPYISED-D------------PERRKYMFCY    173
FWf4/37-421          DTQTEYPDVSDRWMEWCDTNAEAQAFLNTQTYHN---G------------SGRRYYMFCY    171
```

```
MfFcnA/34-418        DTQADYPDVSARWMEWCDTNTEAQAFINSQTYHE---G------------NGRRKYMFCY    171
FFA1/31-415          DTQEDYPDVSERWMEWCDTNPEAQAFIASQDFHE---G------------DGRRKYMFCY    171
FWf2/243-679         PSPNELPDVTNRWKAYCDANY--TTFINSQPYHTGIYNSATGNYVNSEAEFPERKYVFCF    200
Fp279/40-449         SNPASFPDITDRWKEWVDTNPEAQAFIHSQPYHTGIWDAESETYVDASETYPERKYMFAY    188
FWf1/15-439          SNPDYIPNITERWKEWCDTNAEAQAFIASQPYHTGVWDETSKTYINSETEFPERKYMFCY    189
Fp277/26-435         PNPDYIPNITERWKAWVDGSAEAQAFIASQPYHTGTWDADTQSYVDATATYPERKYMFAY    186
SVI_0379/26-367      ANGHWSSKSMTNWAKWVTNKYGSASDDN---------------------------YKKAY    155
Fda1/23-372          DTQVYCSANMNRWRDYVLEQYPSTSLYR---------------------------SFELA    182
Fda2/84-439          DTQVYCSANMNRWRDYVLQQYPSTSLHH---------------------------SFQLG    190
Mef2/22-383          ------IDIKTNWDQFVTQYP----------------------------------SQNDA    139
P5A_FcnA/36-393      ------TDIQAAWDEYYINEWDG--------------------------------DEAAA    144
P19D_FcnA/31-393     ------PEIQDAWDDYVNTNWNG--------------------------------DHGAA    146
                                 *  :

FWf3/213-589         AEYFLKDYALRYGEHFDSWIFDDGATM--EQNGDNATSGVVEEQRIYQAYANAVHAGNPD    225
Fp273/234-617        AEFVLKDYALRYAKYIDSWIFDDGGTM--VENGDNATSGLVEEQRIYQAYANAVHAGNPE    232
FFA2/41-433          AEFVLKEYAMRYGDLIDAWCFDAAHVNM-GAAGDDYSTGDIDTQRVFQAFADACHAGNPD    234
Fhf2/31-439          AEFILKDYAMRYGDLIDAWCFDAAHVNM-ASAGDDYSTGNIDTQRVYQAFADACHAGNPN    234
Fhf1/29-447          AEFILKEYSERYGDLIDAWCFDSADNIM-GACGDEPQSDDVNHQRIYQAFADACHAGNPN    232
FWf4/37-421          AEFILKEYAIRYGDLIDAWCFDSADNIMEDECGDNPDSENVEDQRIYQAFANAVHAGNPN    231
MfFcnA/34-418        AEFILKEYAQRYGDLIDAWCFDSADNVMEDECGDDPASEDVNDQRIYQAFADACHAGNPN    231
FFA1/31-415          AEFILKEYANRYGDLIDAWCFDSADNIMEGECGDDPASDDIDDQRIYQAFAEACHSGNPK    231
FWf2/243-679         AEYILKEYATRYGDLIDAWVFDSGEFM--VSNGDDSESGILEEQRLYEAFANAVHAGNEN    258
Fp279/40-449         AEFVLKEYAVRYGNLIDGWLFDSGDFM--KKNGDNATNGVKAEQQIYKAFADACHAGNPM    246
FWf1/15-439          AEFVLKEYAIRYGDLLDAWCFDSGSWM--GMNGDSQTNGIYEDQMIFNAFKAACHAGNPD    247
Fp277/26-435         AEFVLKVYSERYGDLIDGWLFDSGSFM--YSNGDSATNGVKEDQSLYGAFAKAAQAGNPN    244
SVI_0379/26-367      AEVIIAEYAQRYGSLIDGWWFDHSSCA------------------NIPLLHKITKAANPA    197
Fda1/23-372          MVNIVETLSLRYGSTIDGWWFDHSGFG------------------DSELLHAAALAGNND    224
Fda2/84-439          LVNIVETLSLRYGTLIDGWWFDHSIYG------------------DYNLLPDAARAGNSN    232
Mef2/22-383          YVELLRGFVEVLKDYADGYWLDTVTA-------------------NVEDIVDMIREVDPT    180
P5A_FcnA/36-393      WRNLARGYVERFDGLVDGYWLDNSRNLP-------------G---EVSDFVAMLRSVDPE    188
P19D_FcnA/31-393     WRNLVEGYAKRFKGLVDGFWLDNSKNMA-------------GGQKEIPEFVAMLRDIDPS    193
                        .            *.: :*                                 :

FWf3/213-589         IAIAFNNGRSTVN-----Y--------------------KD--YPFAHAVRFEDFTFGHA    258
Fp273/234-617        IPIAFNNGRSTTA-----Y--------------------AS--TPFAHAVRFEDFTFGHA    265
FFA2/41-433          AAITFNNGIGDRDS----D--------------------PF--VPYVTPSLFEDYKFGHP    268
Fhf2/31-439          AAITFNNGIGDRDS----D--------------------PF--LPYRTPSLFEDYKFGHP    268
Fhf1/29-447          AAISFNNSVGDREL----N--------------------PF-----STATYFDDYTFGHP    263
FWf4/37-421          AAVAFNNSVGDRVE----N--------------------PF-----STATLFDDYTFGHP    262
MfFcnA/34-418        AAIAFNNSVGDREG----N--------------------PF-----TSATLFDDYTFGHP    262
FFA1/31-415          AAISFNNSVGDREG----N--------------------PF-----TTATYFDDYTFGHP    262
FWf2/243-679         AAVAFNNGPNRHEANAANG--------------------TV--TPYSHATHFDDFMFGHP    296
Fp279/40-449         AAISFNNSPNRVT-----E--------------------SL--NPFSEATHYDDYMFGHP    279
FWf1/15-439          AALSFQNSPERDT-----E--------------------EL--NPFSEAVHADDFMFGHP    280
Fp277/26-435         APVSFNNSPERDT-----E--------------------EL--NPFSEATHYDDYMFGHP    277
SVI_0379/26-367      VILAFNKGQKVPLI--------------------------------NNNPGYEDYTSGHP    225
Fda1/23-372          AAVAFNEGDKVPLV--------------------------------NNPETLDDYTFGHP    252
Fda2/84-439          AAVSLNLEGDIFLS--------------------------------NNPEVMEDFTGGHP    260
Mef2/22-383          AVVTTNKGKDYIRLDDGTKAVVASNGVSITGETSPKVGYNIIRHTLDVASTTQDFTNGHV    240
P5A_FcnA/36-393      LTIAVNYDQHYFTDDNGEYLYVDSDGLDDEDE----SDYKIVKHVV--TNEYMDFTNGHV    242
P19D_FcnA/31-393     FAIGVNYETHYFEDEDGNYLKVASDSIDDNDD----REYKIIKHVV--TNEYMDFTNGHV    247
                       :  :                                             *:   **

FWf3/213-589         FGGNNNHAEKI------N--GNQFNLNYRHITRMTETNGLVHAG---------------    294
Fp273/234-617        FGGNNNHAEKE------N--GNQFNNNYRHVTRMTETNGYVHAG---------------    301
FFA2/41-433          FGGAGNMVEPR---------DPLYTVNFGICEYMRDTNGHPYTN---------------    303
Fhf2/31-439          FGGAGNMVEPR---------DPLYTVNFGICEYMRDNNGLPYTN---------------    303
Fhf1/29-447          FGGAGNMVEPR---------DPLYTYNYHVIEWMSDYNGSAFLVESVNKEISTDGNKILI    314
FWf4/37-421          FGGAGNMVEN----------ETLYRYNYHVIEWMQDYAGDAFLD---------------    296
MfFcnA/34-418        FGGAGNMVVP----------EALYTYNHDLVVFMQTNNGYAFRD---------------    296
FFA1/31-415          FGGAGNMVEN----------ETLYGYNFGVIEWMDDYNGSAFLD---------------    296
FWf2/243-679         YNGGKSIGKDKADYYDTGAYPTNYGRNYAHMLWMQGTGGNVHLYR-D--------SSNPV    347
Fp279/40-449         YNGGTFIGNHD------N---TNYDRNYRHIQKVSETNGNIHSGSLD--------AGTDS    322
FWf1/15-439          YNGGRDGGSHT------IGTPSLYSRNYAHIEKMKETNGYAHR------------GSDP    321
Fp277/26-435         YNGGRVIGNHE------N---GLYERNYAHIQKITATNGNVHSGSVA--------AGTDA    320
SVI_0379/26-367      TPLRRSTVS-ND------------NLP---MVESIEASSNGYV----------------    252
Fda1/23-372          TPIGSEVSSDDK------------NLP---MLTSIEATLDGIL----------------    280
Fda2/84-439          TPIARVVSSDDT------------NLP---MLTAIEDAPNGIF----------------    288
Mef2/22-383          TSII-KAPTDSW------------GYEEFTVGDMIEEPYTE------------------    268
P5A_FcnA/36-393      TPLGRGAPPNSW------------AYEEYTIPDMIEVPWET------------------    271
```

```
P19D_FcnA/31-393      TPMGQGAPPNSW-----------GYEEYTIPHMIEKPWDS------------------    276


FWf3/213-589          GNWDWDDKIVGNFHSKLSTTAWKYGPN-QAWEQADFNQWNLEAIQAGGSMTWGGSFNRA-    352
Fp273/234-617         GNWEWDDRIVGNFHSKLSTTAWTYGPN-QAWEEADFLQWNLEAMHAGGMMSWDGSRPRT-    359
FFA2/41-433           DDIDWNDNVVAHFFPKQSTTSWNDGGT-PCLTDEEFVEWNNVGLINGGGITWGTPLVITN    362
Fhf2/31-439           DGIDWNDNVVAHFFPKQSTTSWNDGGT-PCLTDGEFVEWNNVGLINGGGITWGTPLVITN    362
Fhf1/29-447           ENRAWNDNVVSHFFPKQSATSWNAGNT-PCLTDEEFVEWTSTGIIDGGAITWGTPLVRTN    373
FWf4/37-421           DTRDWNDKVVSHFFPKQSSTSWNAGST-PCLTDEQFVEWTTNGIIDGGAITWGTPLIRTN    355
MfFcnA/34-418         DTRTWNDNVVAHFFPKQSTTSWNAGNT-PCLTDEQFVEWTSTGIVNGGGITWGTPLVRTN    355
FFA1/31-415           DDRTWNDNVVSHYFPKQSATSWNAGNT-PCLTDDQFVEWTSKGIIDGGAITWGTPLIRTN    355
FWf2/243-679          DEWEFDNKVVGGFYPPMSTTSWQGGAT-QALEQVDFELWNQVAIDHGGTIIWGTALEIYN    406
Fp279/40-449          QNWDWDDKVVGHFDPPMSRTRWNSGAD-PGLSDEDFLLWNYESAVGGGAISWGAPLYSP-    380
FWf1/15-439           QTWTWDDNVVAHYDPPMSTTSWNGGNT-PALTDEEFNLWNLEAVQNGGAISWGLPLVKK-    379
Fp277/26-435          HDWDWDDKVIGHFDPPMSTTSWNGGNT-PALTDDEFNLWNLESAKGGGAISWGLALVGK-    378
SVI_0379/26-367       D-DGAGHKSLGHMFMPLG-NTWNSGL--SVWPLSKAVDWITRVNNACGAWTWNVATQDTN    308
Fda1/23-372           TGSGDDVGSVGHMFMPLQ-ESWNGGT--VVFSEAKGSDWLNRALKAGGAFTWALSQDSND    337
Fda2/84-439           TGTGDDVDALGHMFLPLQ-ETWNGGT--VVFSEAKGTEWLNRVTRAGGALTWALSHEG--    343
Mef2/22-383           --YLGNP-VIKHAWFPVR-YRWHTSTRELLYDTERSYRTVKSIIDAGASVTLANTVDYNT    324
P5A_FcnA/36-393       --YDGSKYALKHGWFPIR-NSWSGSKAELMFDVEQAYRFVRTVTDGGAAMTWSTTQ--DN    326
P19D_FcnA/31-393      --VDGNHYALMHGWFPIR-FSWSGSGAELMFETEQAYRFVRTITDGGAAMTWSTTQ--KK    331
                           .   :           *  .                       .

FWf3/213-589          ------ETAI-YDWVYVLLEGLDDYLVQYGNP----------------      377
Fp273/234-617         ------SDTL-YTWAYDLLKGLDDHLAKYQKP----------------      384
FFA2/41-433           LNNASANLTL-QPYALRQLELVDADLSANQFP----------------      393
Fhf2/31-439           LNNASPNLTL-QPYALRQLELVDADLSVNQYPGAPNWARQYTVLPEII      409
Fhf1/29-447           LNN-APDLTL-QPYALRQLALTDEYLKQNQFPGAPNWARQTTRLSEVI      419
FWf4/37-421           LEN-SPELVL-RDYALTQLTLTDDFLKEFQYP----------------      385
MfFcnA/34-418         LEN-APVLTL-QPYALNQFELTDTYLKEFQSP----------------      385
FFA1/31-415           LEN-SPVLTL-RDYAVTQFELTDAYLKEFQSP----------------      385
FWf2/243-679          LNNANPSLIA-RDWAIAQIQGADDYFASLYNS----------------      437
Fp279/40-449          PG-TGEQLLI-REWGMAQLELIDADLAERQEP----------------      410
FWf1/15-439           SG-TNEQLVG-TDWALAQLNGMDAHLMELEAPGAPNWSRQETVLSEAK      425
Fp277/26-435          SSNDNAKLVA-TDWALAQLTGMDAHLAQLQEP----------------      409
SVI_0379/26-367       SKLADHAITFMNDVTAKIISNGGTRCTVTIDPS---------------      341
Fda1/23-372           ELGGGGARLI-SE------------------P----------------      350
Fda2/84-439           SVSGGEAMLI-SA------------------P----------------      356
Mef2/22-383           GRMMPDEMVMMKEVNRRLLL--NEDCEAYTRPAGARLEGE--------      362
P5A_FcnA/36-393       GYMTADEMSIMIEISNRMTQTPKPDYSVYERP----------------      358
P19D_FcnA/31-393      GYMSADEMDIMIEINNRMTQAPKLDYEAYERP----------------      363
```

Figure S1. Multiple alignment of the catalytic domain (D1) of the fucoidanases Mef2 with other GH107 family members. The conserved catalytic amino acid aspartate (red) and histidine (blue) are indicated. Conserved amino acids around the -1 subsite are indicated purple. Changes in conserved positions (orange and underlined) are indicated. Mef2 (GenBank: ON099398), Fhf1 (RefSeq: WP_066217780), Fhf2 (RefSeq: WP_066217784.1), Fhf3 (RefSeq:WP_066217796.1), FFA1 (RefSeq: WP_057784217.1), FFA2 (RefSeq: WP_057784219.1), Fda1 (GenBank: AAO00508.1), Fda2 (GenBank: AAO00509.1), MfFcnA (GenBank: CAI47003.1), P5AFcnA (GenBank: AYF59291.1), P19DFcnA (GenBank: AYF59292.1), SVI_0379 (GenBank: BAJ00350.1), FWF1 (GenBank: ANW96097.1), FWF2 (GenBank: ANW96098.1), FWF3 (GenBank: ANW96115.1), FWF4 (GenBank: ANW96116.1), Fp273 (GenBank: AYC81238.1), Fp277 (GenBank: AYC81239.1), Fp279 (GenBank: AYC81240.1).

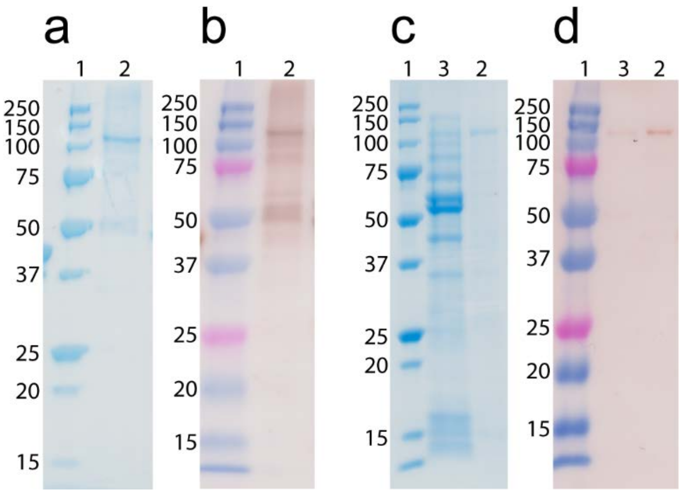

Figure S2. Recombinant expression and purification of the Mef2 fucoidanase. SDS-PAGE of a) purified Mef2 (2)(expression at 20 °C, O/N) and c) crude (3) and purified Mef2 (2)(expression at 37 °C, 4h). Western blot of b) purified Mef2 (2)(expression at 20 °C, O/N) and d) crude (3) and purified Mef2 (2)(expression at 37 °C, 4h). 1) Protein standards.

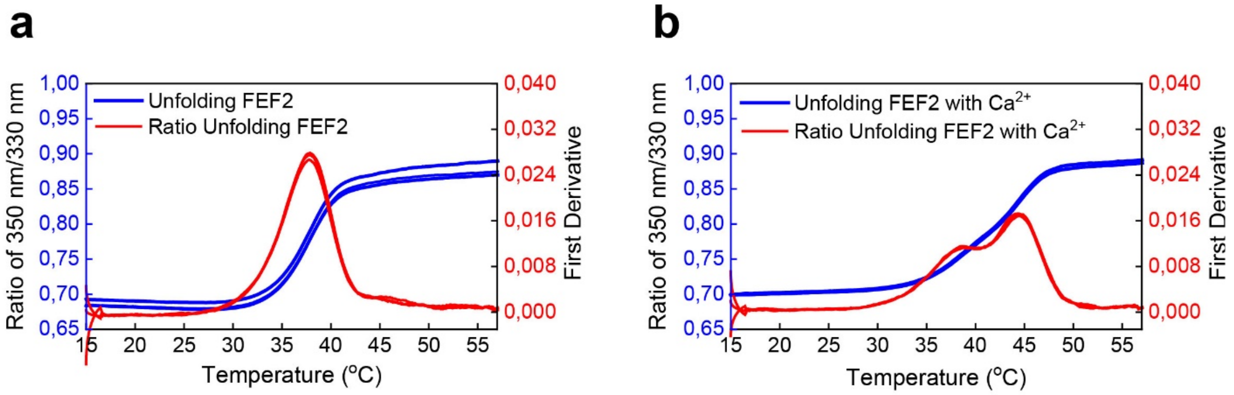

Figure S3. Thermostability assessments of Mef2 by DLS. a) Thermostability of Mef2 without Ca^{2+} and b) with Ca^{2+}.

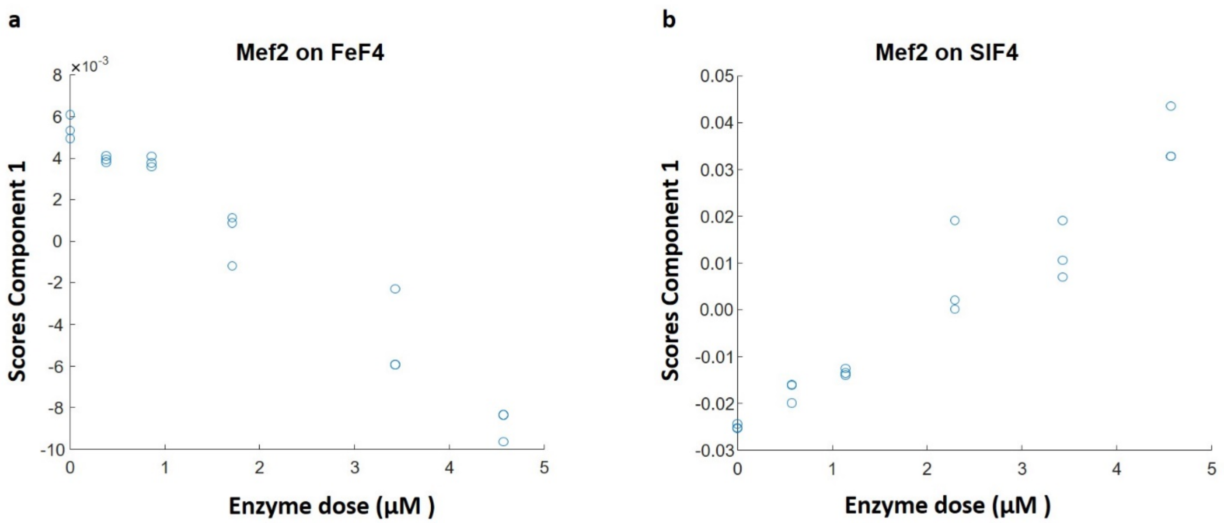

Figure S4. PARAFAC first component scores versus enzyme dosage were plotted to build calibrations. a) for Mef2 on *F. evanescens* FeF4 fucoidans with R^2=0.97, b) for Mef2 on *S. latissima* SlF4 fucoidans with R^2=0.98.

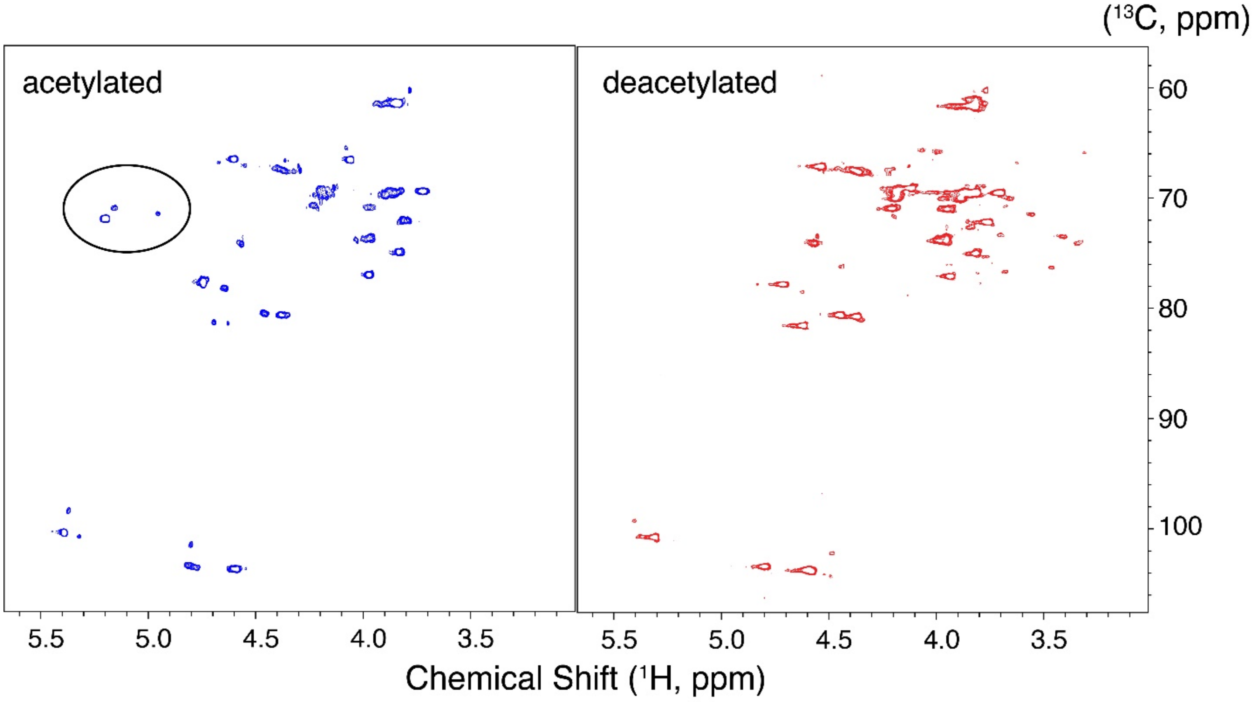

Figure S5. NMR analysis of deacetylation of *S. latissima* fucoidans. Circle indicates acetylations, which were gone after the deacetylation process.

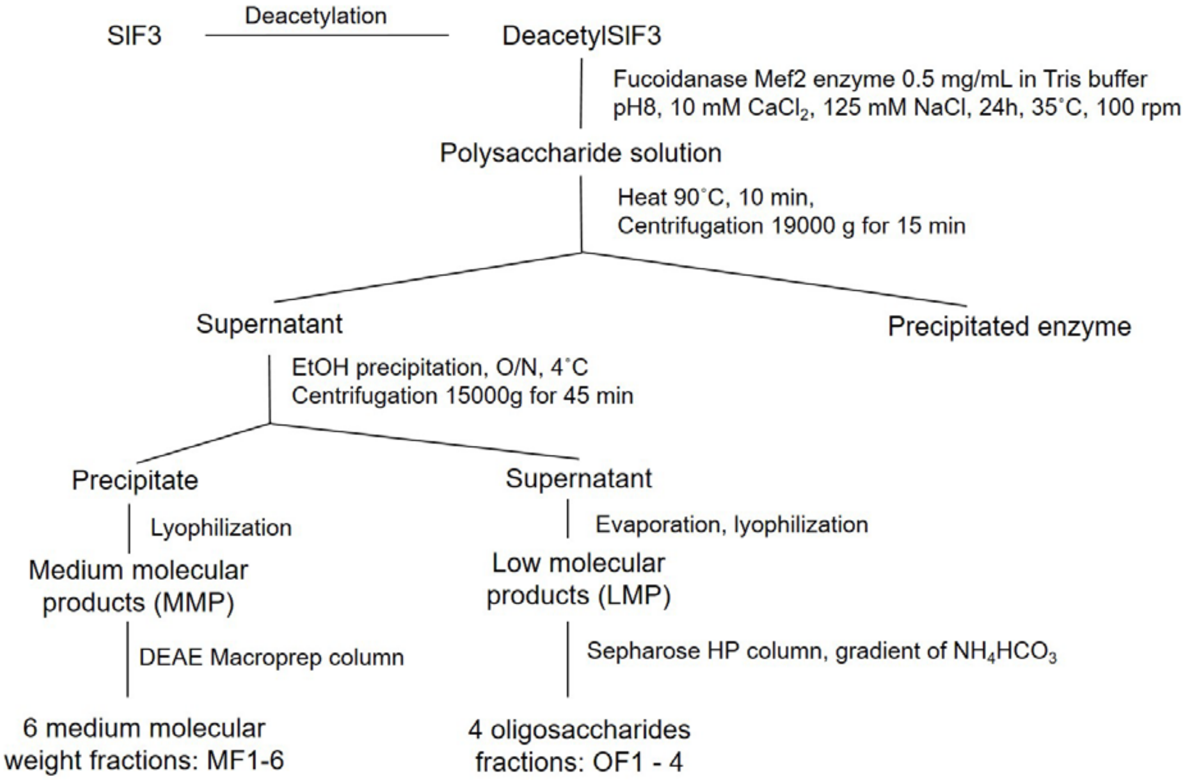

Figure S6. Flow-chart of enzymatic hydrolysis of fucoidans from *S. latissima* and further product separation

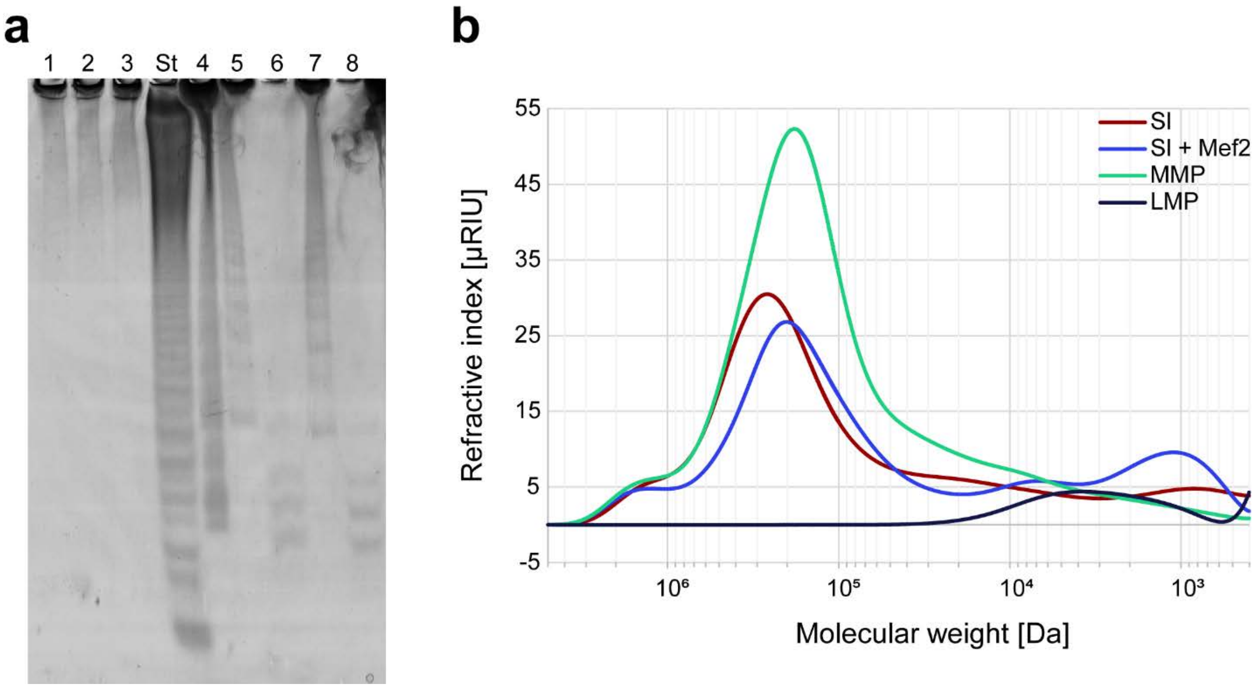

Figure S7. Medium and low molecular weight fucoidans after Mef2 hydrolysis of fucoidans from *S. latissima*. a) 1) DeSlF3; 2) DeSlF3 + heat 90 °C, 10 min; 3) DeSlF3 + heat 90 °C, 1 hour; 4) DeSlF3 + Mef2; 5) MMP from DeSlF3 + Mef2; 6) LMP from DeSlF3 + Mef2; 7) MMP (DeSlF3 + Mef2) + Mef2; 8) LMP (DeSlF3 + Mef2) + Mef2. The standard (St) is the product profile of FFA2 treatment of fucoidan from *F. evanescens*. b) HP-SEC chromatograms of MMP and LMP of Mef2 on fucoidan from *S. latissima.*

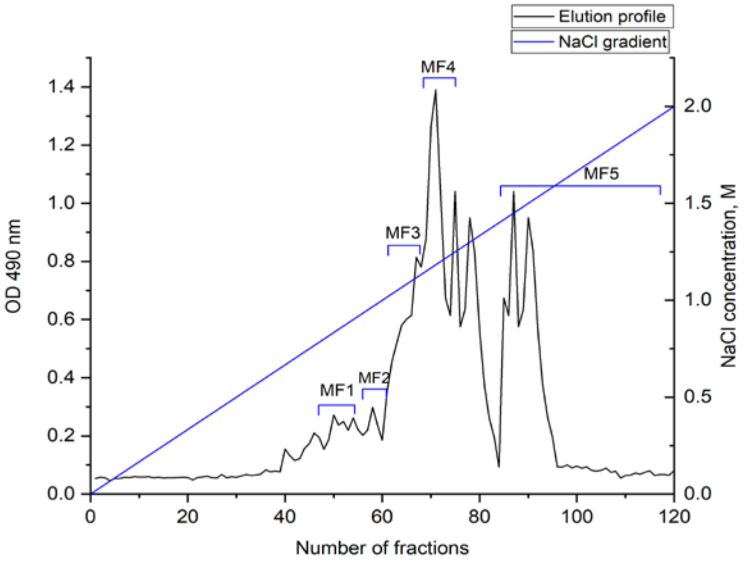

Figure S8. Separation of *S. latissima* MMP products on DEAE macroprep column. The total sugar content was detected by the phenol-sulfuric acid method.

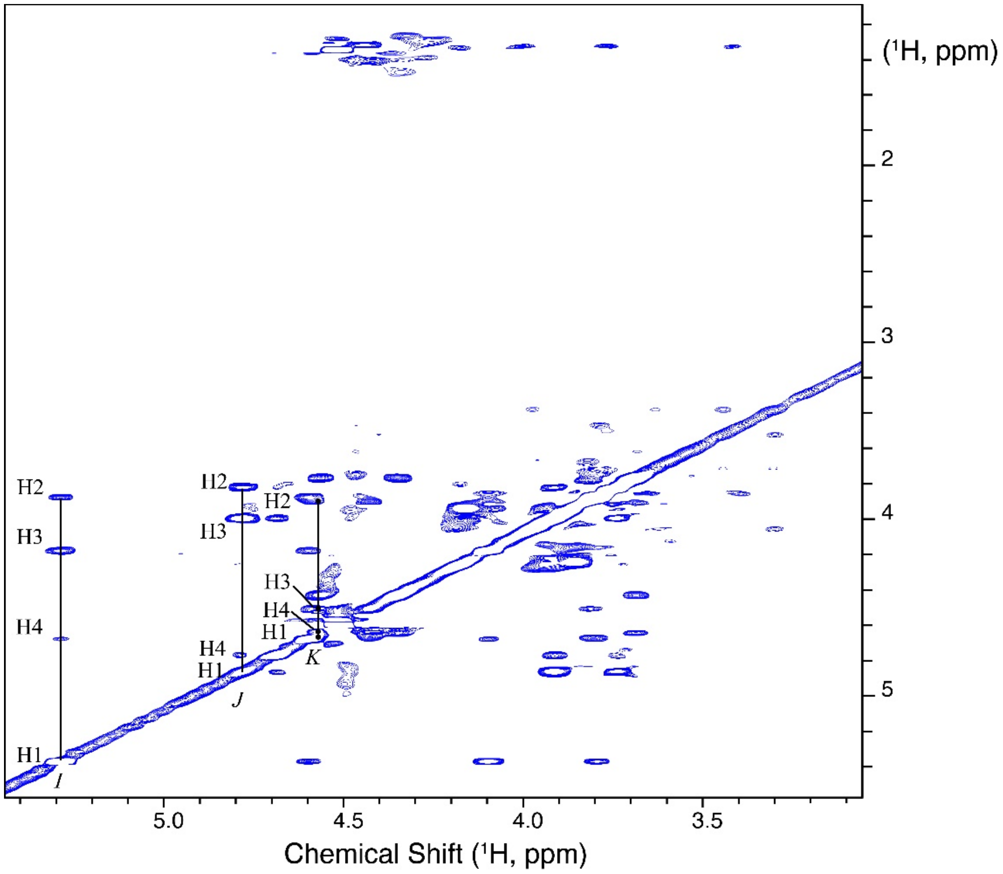

Figure S9. ¹H-¹H TOCSY NMR spectrum for the deacetylated *S. latissima* MMP. The spectrum shows the correlations between H1, H2, H3 and H4 for the three spin systems I, J and K in the MMP described in Figure 7d and in Table 3.

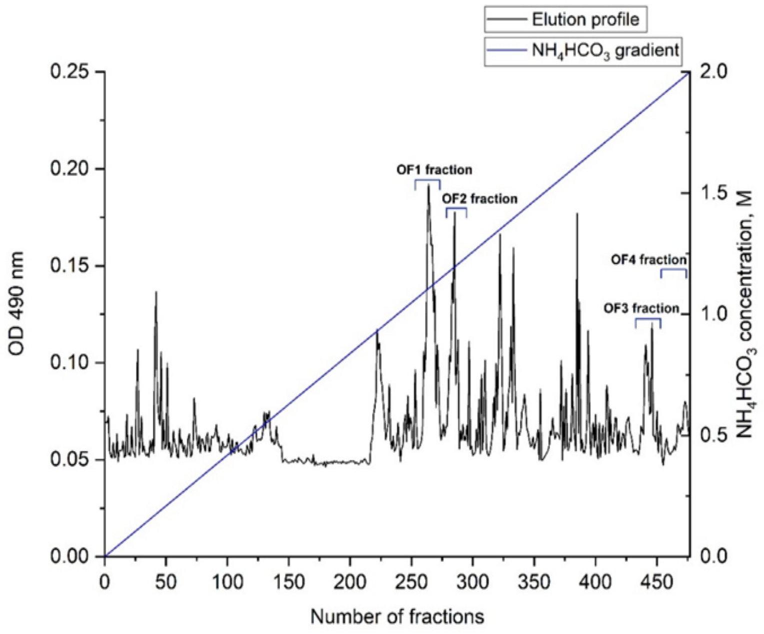

Figure S10. Separation of *S. latissima* LMP products on Q sepharose High Performance column. The total sugar content was detected by the phenol-sulfuric acid method.

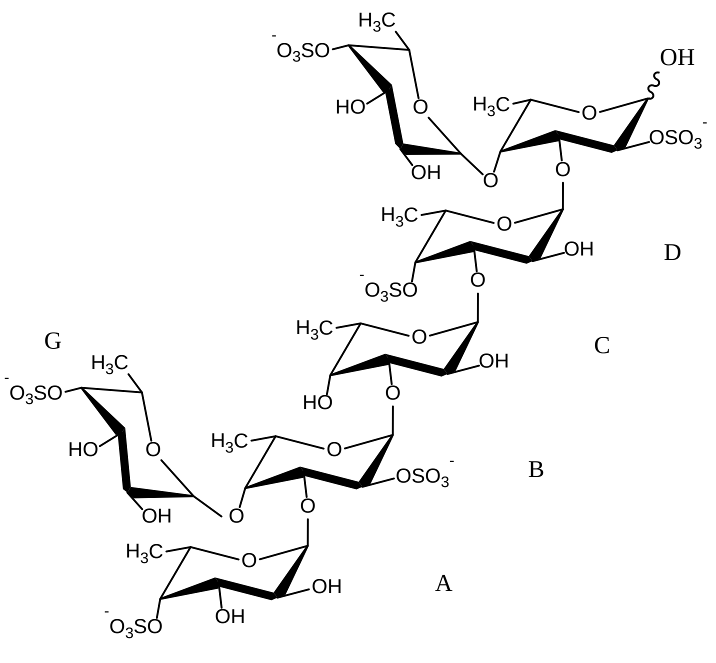

Figure S11. The proposed structure of the *S. latissima* OF2 fraction

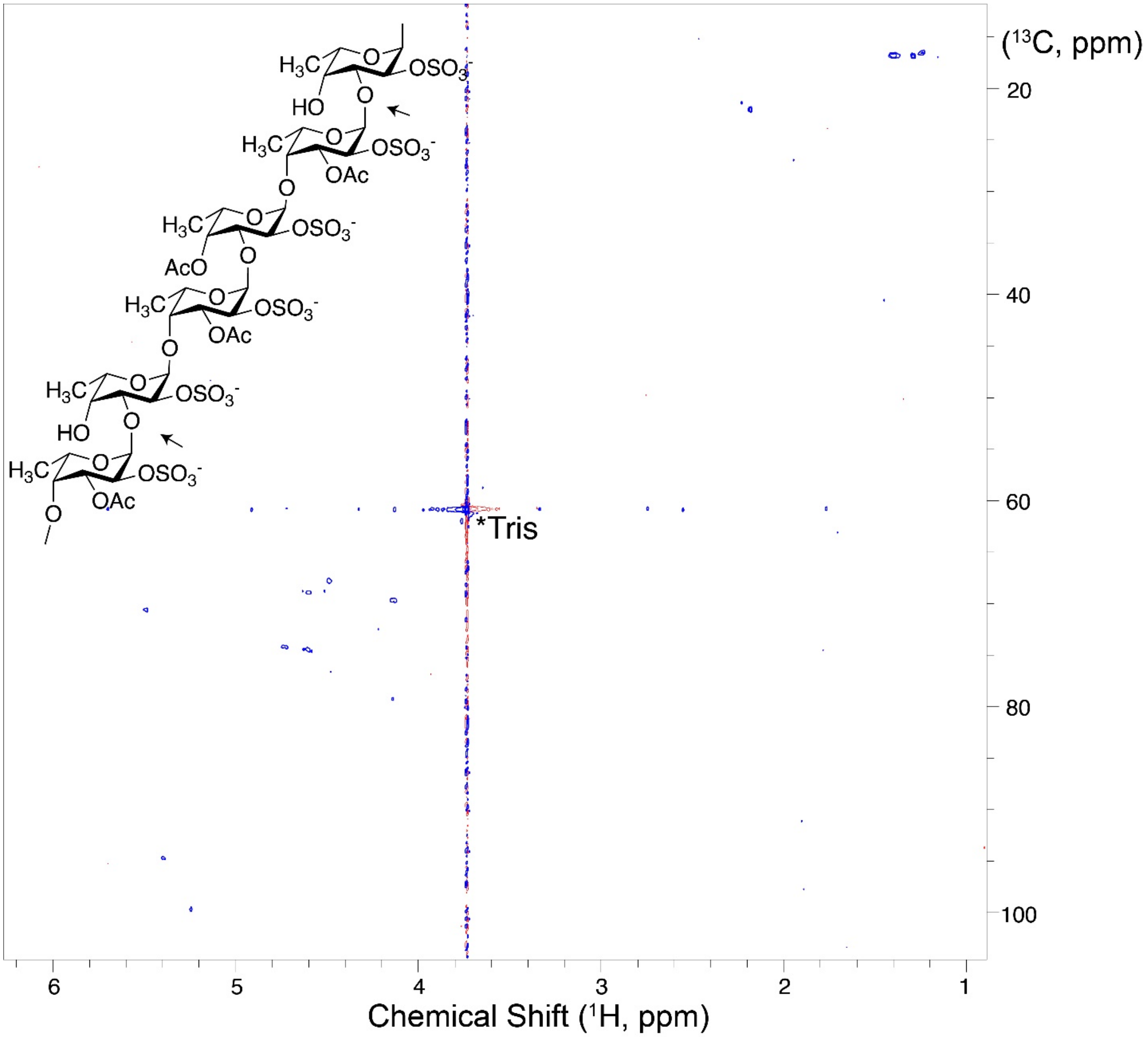

Figure S12. ^{1}H-^{13}C NMR spectra of LMP from *F. evanescens* fucoidan after Mef2 hydrolysis. Upon cleavage of *F. evanescens* fucoidans, the reducing end is not visible due to limitations with regards to material and due to length of the product. Arrows indicate possible Mef2 cleavage sites.

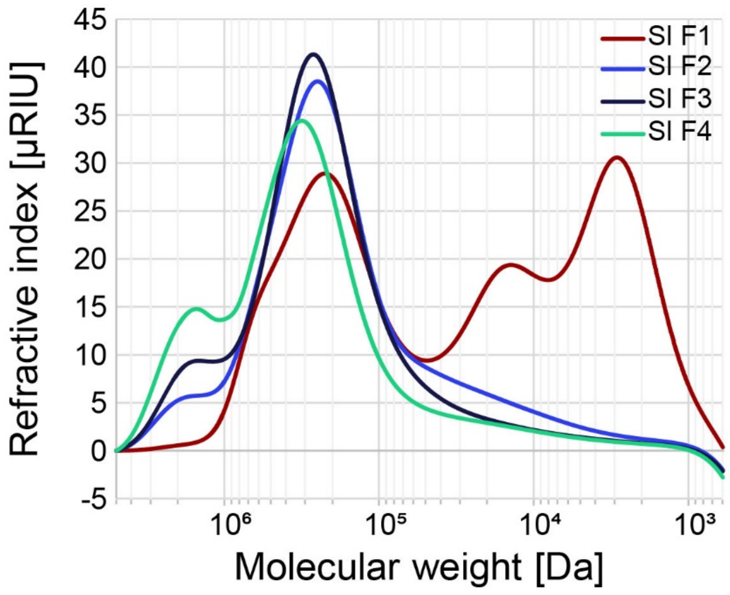

Figure S13. HP-SEC chromatogram of fucoidan fractions from *S. latissima* from Ocean Rainforest after IEX purification. The SlF1 fraction contains low molecular weight compounds (likely partially degraded alginates) of around 2 - 12 kDa and a proportion of HMP compounds between ~200–600 kDa. The SlF2 and SlF3 contain HMP fucoidan compounds ranging from ~250 kDa to over 800 kDa. The SlF4 contain more HMP compounds ranging from ~300 kDa to over 800 kDa. Pullulan was used as standard.
